# Chemoembolization Plus Radiotherapy versus Chemoembolization Plus Sorafenib for the Treatment of Hepatocellular Carcinoma Invading the Portal Vein: A Propensity Score Matching Analysis

**DOI:** 10.3390/cancers12051116

**Published:** 2020-04-29

**Authors:** Hee Ho Chu, Jin Hyoung Kim, Ju Hyun Shim, Sang Min Yoon, Pyeong Hwa Kim, Ibrahim Alrashidi

**Affiliations:** 1 Department of Radiology and Research Institute of Radiology, Asan Liver Center, Asan Medical Center, University of Ulsan College of Medicine, 88, Olympic-Ro 43-Gil, Songpa-Gu, Seoul 05505, Korea; chuzzang1224@gmail.com (H.H.C.); peace4701@hotmail.com (P.H.K.); dr.ialrashidi@gmail.com (I.A.); 2 Department of Gastroenterology; Asan Liver Center, Asan Medical Center, University of Ulsan College of Medicine, 88, Olympic-Ro 43-Gil, Songpa-Gu, Seoul 05505, Korea; s5854@amc.seoul.kr; 3 Department of Radiation Oncology, Asan Liver Center, Asan Medical Center, University of Ulsan College of Medicine, 88, Olympic-Ro 43-Gil, Songpa-Gu, Seoul 05505, Korea; drsmyoon@amc.seoul.kr

**Keywords:** hepatocellular carcinoma, portal vein tumor thrombosis, transarterial chemoembolization, sorafenib, radiotherapy

## Abstract

A combination of transarterial chemoembolization (TACE) plus sorafenib or radiotherapy (RT) has demonstrated efficacy in patients with advanced hepatocellular carcinoma (HCC). Here, the two combined treatment approaches were compared in patients with HCC and portal vein tumor thrombus (PVTT). Data from 307 patients treated with TACE plus RT (*n* = 203) or TACE plus sorafenib (*n* = 104) as first-line treatment for HCC with PVTT were retrospectively evaluated. Using the propensity model to correct selection bias, 87 patients were included from each treatment group. During follow up (median, 12 months) in the entire study population, the median progression-free survival (PFS) and overall survival (OS) were significantly longer in the TACE plus RT group than in the TACE plus sorafenib group (6.5 vs. 4.3 months, respectively; *p* = 0.017 and 16.4 vs. 12 months, respectively; *p* = 0.007). Following propensity score matching, the median PFS and OS in the two groups showed no statistically significant difference. Multivariable analysis found no significant association between PFS or OS and the treatment type. In conclusion, this retrospective study of data from patients with advanced HCC with PVTT shows that PFS and OS did not differ significantly in patients treated with TACE plus RT and TACE plus sorafenib.

## 1. Introduction

Advanced hepatocellular carcinoma (HCC; Barcelona Clinic Liver Cancer (BCLC) stage C) describes patients who have symptoms and/or vascular invasion or extrahepatic spread. Systemic therapy, such as sorafenib, has emerged as a promising treatment approach for these patients, although the survival benefit is limited to < 3 months [1,2]. Macroscopic vascular invasion of HCC was shown to be a significant prognostic factor for poorer overall survival (OS) in two randomized controlled trials of sorafenib [3]. Macroscopic vascular invasion is associated with a median survival of only 2–4 months if left untreated [4,5,6]. Previous studies have demonstrated that repeated conventional transarterial chemoembolization (TACE) is associated with significant survival benefits in patients with HCC invading the portal vein when compared with supportive care [7,8,9]. However, to date, the reported OS period for HCC patients with portal vein tumor thrombosis (PVTT) who receive TACE is slightly better than that of patients who receive sorafenib therapy (5.9 vs. 4.4 months, respectively; *p* = 0.003) [10], although this observation requires validation in a prospective study.

TACE-induced hypoxia in surviving tumor cells leads to the release of angiogenic growth factors, which can contribute to tumor recurrence or metastases and impact on outcomes. Sorafenib inhibits tumor progression by blocking angiogenic growth factors, and a combination of TACE and sorafenib may, therefore, be beneficial. The recent phase III STAH [11] and TACTICS [12] trials showed that this combination regimen could improve clinical outcomes, specifically delaying tumor progression, in comparison with sorafenib [11] or TACE [12] monotherapy in patients with unresectable HCC. Another combination strategy is TACE plus radiotherapy (RT), with the rationale being that reducing PVTT with RT can delay intravascular tumor growth and the deterioration of liver function by preserving adequate portal flow as well as by facilitating subsequent treatment of the primary HCC [13]. Previous retrospective studies and a recent randomized controlled trial have shown a clear survival benefit with this approach when compared with sorafenib or TACE monotherapy for the treatment of HCC with macrovascular invasion [10,14,15]. However, it is not clear which combined strategy is better in the palliative treatment of HCC with PVTT. The aim of this retrospective study was to compare the effectiveness of the two combined strategies in the treatment of patients with HCC with PVTT.

## 2. Materials and Methods

### 2.1. Patients

HCC was diagnosed according to the American Association for the Study of Liver Diseases (AASLD) or European Association for the Study of the Liver (EASL) criteria [16,17]. The presence and extent of PVTT were evaluated using computed tomography (CT) or magnetic resonance imaging (MRI), and were confirmed by detecting the enhancement of an intraluminal mass expanding into the portal vein (PV) on the arterial phase and a low-attenuation intraluminal mass on the portal phase [14,15].

TACE was recommended in patients with HCC invading the PV who had Child–Pugh class A or B liver function and an Eastern Cooperative Oncology Group (ECOG) performance status of 0–2. TACE was contraindicated in patients with HCC invading the PV if they had Child–Pugh class C liver function, a total bilirubin level ≥ 3 mg/dL, serum creatinine levels ≥ 1.5 mg/dL, tumors occupying > 50% of the liver, or an ECOG performance status of 3 or 4. Extrahepatic metastasis was not a contraindication for TACE.

Patients who received combined treatment with TACE plus RT, or TACE plus sorafenib as first-line treatment for HCC with PVTT between January 2014 and September 2019 were included in the analysis. A treatment decision on which option would be better for patients with HCC and PVTT among these two combined treatment options was usually made by our multidisciplinary team. Physicians explained the two treatment options to all patients, and a final treatment decision was determined for each patient after consideration of the physician’s and patient’s preferences, as well as the cost.

The study design was approved by the institutional review board of Asan Medical Center (2020-0490), and the requirement for patient consent was waived because of the retrospective nature of the analysis.

### 2.2. Transarterial Chemoembolization

The TACE procedure was performed as described previously [8,9,10,14]. Cisplatin-based TACE was undertaken using a cisplatin dose of 2 mg/kg of body weight. Using a microcatheter, an emulsion of iodized oil (Lipiodol^®^, Guerbet, Roissy, France) of 3–20 mL and cisplatin in a 1:1 ratio was infused into the lobar, segmental, or subsegmental feeding artery until portal venules around the tumor were visualized by iodized oil filling (oily portogram sign). This was then followed by embolization with Gelfoam slurry (Upjohn, Kalamazoo, MI, USA) until near stasis of arterial flow was achieved [9]. If there was a significant arterioportal shunt, embolization with Gelfoam slurry was first performed to occlude the shunt, after which the iodized oil/cisplatin emulsion was infused, and embolization with Gelfoam slurry was performed [9]. TACE was repeated every 4–6 weeks if residual viable tumor tissue was evident on follow-up CT scans without deterioration of hepatic function.

### 2.3. External Beam Radiation Therapy

RT was performed as described previously [13,15,18]. In patients in the combined TACE and RT group, RT for PVTT began within 3 weeks of the first TACE. Four-dimensional CT simulation (GE LightSpeed RT16; GE Healthcare, Waukesha, WI, USA) was performed during free breathing in all patients. The four-dimensional CT images synchronized with the respiratory data were sorted into 10 CT series based on the respiratory phase (Advantage 4D version 4.2; GE Healthcare). A real-time position management gating system (Varian Medical Systems, Palo Alto, CA, USA) was used for the analysis of patients’ respiration. Gross tumor volume (GTV) included the PVTT and a 2 cm margin into the contiguous HCC at the end-expiratory phase of the CT image for huge, multiple, or infiltrative HCC. In patients with small HCC, which can be covered by a radiation port, the GTV included both the entire HCC and PVTT. Internal target volume (ITV) was delineated as the sum of the individual GTVs, as defined within the gated respiration phases. Planning target volume (PTV) was expanded to include a 0.7 cm margin from the ITV. A three-dimensional conformal radiation therapy technique was used to determine target volumes, radiation ports, and dose prescriptions using a planning system (Eclipse, version 10.0; Varian Medical Systems), and the actual beam delivery was performed with a respiratory-gated beam delivery technique. The dose per fraction to the PTV was 2–5 Gy using 6 or 15 MX-rays at five fractions per week with a linear accelerator (Varian Medical Systems). The total dose was determined by the normal liver volume, baseline hepatic function, and the maximum dose to the surrounding normal organs according to the previous dose prescription guidelines [13,15,18].

### 2.4. Sorafenib Therapy

Patients in the combined TACE and sorafenib group started sorafenib 400 mg BID on day 4 after the first TACE. Thereafter, sorafenib was administered on an interrupted schedule, with an interval of 4–7 days before and after each subsequent TACE [19]. Sorafenib doses were reduced or delayed, or sorafenib was temporarily interrupted if there was clinically significant toxicity (grade 2 or higher according to National Cancer Institute Common Terminology Criteria for Adverse Events, version 4.0) [19,20]. Dose escalation or rechallenge with sorafenib was determined when toxicity decreased and the patient could tolerate the medication well [19].

### 2.5. Definition and Data Assessments

PVTT was classified as previously described [10,20], as follows: type A, PVTT in the main portal vein; type B, PVTT in the first-order portal vein branch (the right or left portal vein); and type C, PVTT in the second- or lower-order portal vein branches (segmental branches of portal vein or higher).

**Figure 1:**
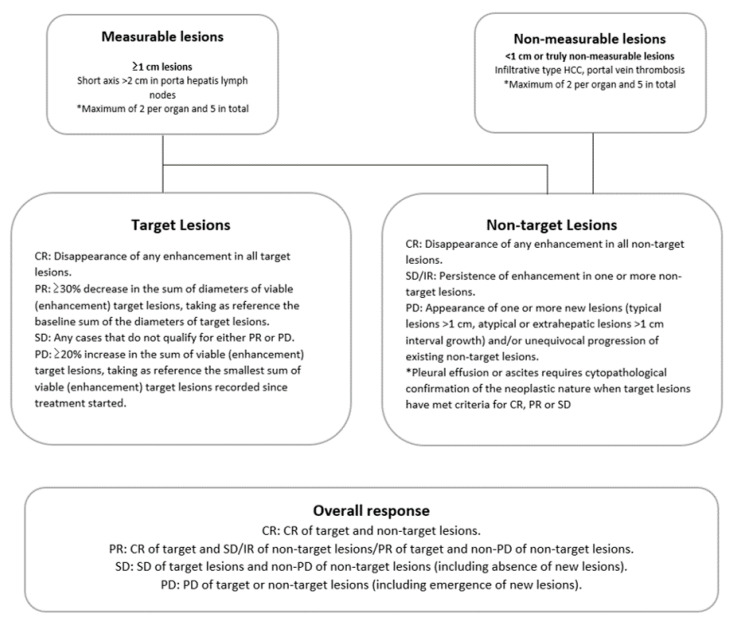
Response evaluation in hepatocellular carcinoma (HCC) by the modified Response Evaluation Criteria in Solid Tumors (mRECIST) guidelines (adapted from [22]).

Radiologic response was evaluated every 4–6 weeks using dynamic CT or MRI, according to the modified Response Evaluation Criteria in Solid Tumors (mRECIST) guidelines [21,22]. Radiologic response was dichotomized as objective response, including complete response (CR) and partial response (PR), or non-regression, including stable disease (SD) and progressive disease (PD) (Figure 1). Radiologic response was evaluated by the consensus of two radiologists, who were blinded to patient demographic information and patient outcome. The best overall response during treatment was categorized as the final response [19].

Progression-free survival (PFS) and OS rates were calculated using the Kaplan–Meier method and compared using the log-rank test. PFS was defined as the time elapsed between treatment initiation and tumor progression (on the basis of the mRECIST guidelines) or death from any cause [23]. The OS periods were measured in months from the time of initial treatment to a patient’s death. Survival calculations were censored at the time of surgical resection owing to downstaging after repeated treatment or for a palliative purpose in some patients.

The groups were compared using Student’s *t*-test for continuous data and the chi-square test for categorical data. Major complications (serious adverse events; grade 3 or 4 toxicities) were evaluated and compared using National Cancer Institute Common Terminology Criteria for Adverse Events, version 4.0) [19,20]. Multivariable Cox proportional hazards modeling with backward elimination method was performed to identify the independent predictive function of the treatment type (TACE + sorafenib/TACE + RT) with regard to the PFS and OS. Only variables associated with a *p*-value < 0.05 in the univariable Cox analysis were entered into the multivariable model. Statistical analyses were performed using computer statistical software SPSS, version 21 (IBM Corp., Armonk, NY, UK), and two-sided *p*-values < 0.05 were considered statistically significant.

To minimize the effects of potential confounders and selection bias, propensity score matching was conducted. The psmatch2 macro in Stata 11.0 software (StataCorp LP, College Station, TX, USA) was used, and one-to-one matching between the two groups was accomplished by using the nearest-neighbor matching method, as described previously [24]. Independent variables entered into the propensity model included age; sex; etiology; ECOG performance status; Child–Pugh score; presence or absence of extrahepatic metastasis; maximal tumor size; tumor number; serum bilirubin, albumin, and alpha-fetoprotein (AFP) levels; tumor type; tumor extent; extent of PVTT; and presence or absence of combined hepatic vein invasion. After adjustment for these factors, PFS and OS rates and tumor responses were recalculated for the two groups.

## 3. Results

### 3.1. Patients

During the study period, 307 patients received TACE plus RT (*n* = 203) or TACE plus sorafenib (*n* = 104) as first-line treatment for HCC with PVTT. The baseline characteristics of these patients are summarized in Table 1. Both groups had a male predominance and a similar mean patient age. Patients with chronic hepatitis B virus infection were more prevalent in the TACE plus RT group than in the TACE plus sorafenib group (92.6% vs. 78.8%, respectively), although chronic hepatitis C virus carriers were more common in the TACE plus sorafenib group than in the TACE plus RT group (8.7% vs 3%, respectively; *p* = 0.002). Total bilirubin and AFP levels did not differ between the two groups, although the maximal tumor size was larger in the TACE plus sorafenib group (mean, 10.6 vs. 9.2 cm in the TACE plus RT group; *p* = 0.003). Serum albumin level was significantly lower in the TACE plus sorafenib group. The proportion of patients with each ECOG performance status and Child–Pugh score, tumor type (nodular/infiltrative), tumor involvement (unilobar/bilobar), extent of PVTT, and combined hepatic vein invasion was similar in both groups. After propensity score matching, the baseline characteristics were more balanced than they were before, and no statistically significant differences were seen between the two groups (Table 1).

### 3.2. Radiologic Response after Treatment

**Figure 2:**
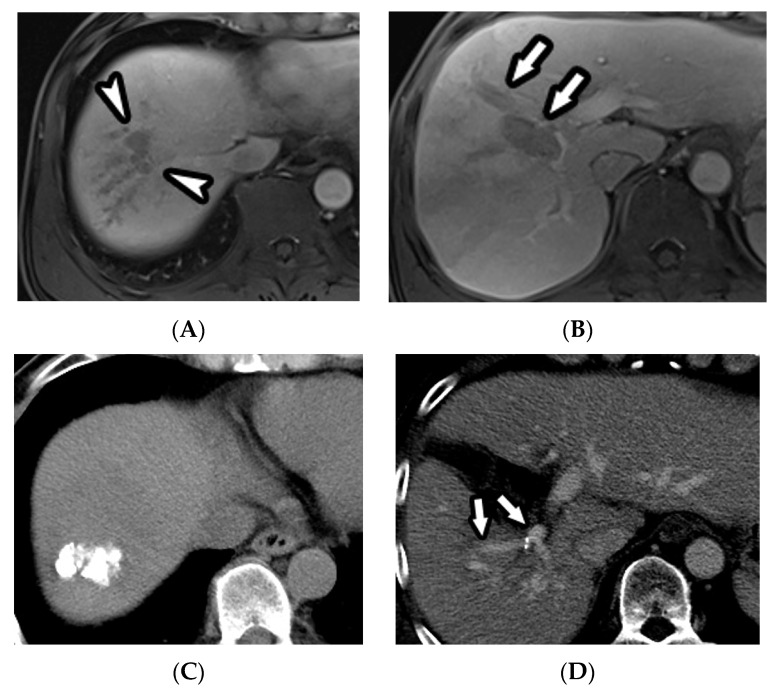
(**A**) A 51-year-old male patient with HCC and lobar PVTT. (**A**,**B**) Contrast-enhanced axial MRI images in the venous phase show a small HCC (3.4 cm maximum diameter, arrowheads) with right lobar PVTT (arrows). (**C**,**D**) Six months after TACE plus RT and additional TACE, non-contrast enhanced (**C**) and contrast-enhanced axial CT (**D**, in the venous phase) images show a compact lipiodol uptake in the tumor (**C**) and disappearance of the right lobar PVTT (**D**, arrows), with a decrease in volume of the right hemiliver.

During follow up (median, 12 months; range, 1.4–70.8 months), 49 patients (24.1%) showed a CR in the TACE plus RT group (Figure 2), 70 (34.5%) showed a PR, 28 (13.8%) progressed, and 56 (27.6%) had SD. In the TACE plus sorafenib group, 18 showed a CR (17.3%) and 25 (24%) a PR, while 19 (18.3%) progressed and 42 (40.4%) had SD. The objective tumor regression (CR or PR) rates were 58.6% and 41.3% in the TACE plus RT and TACE plus sorafenib groups, respectively (*p* = 0.005). After propensity score matching, 19 patients (21.8%) showed a CR, 29 (33.3%) showed a PR, 14 (16.1%) progressed, and 25 (28.7%) had SD in the TACE plus RT group. In the TACE plus sorafenib group, 15 showed a CR (17.2%) and 22 (25.3%) a PR, while 14 (16.1%) progressed and 36 (41.4%) had SD. The objective tumor regression (CR or PR) rates were 55.2% and 42.5% in the TACE plus RT and TACE plus sorafenib groups, respectively (*p* = 0.095).

### 3.3. Progression-Free Survival Analyses

**Figure 3:**
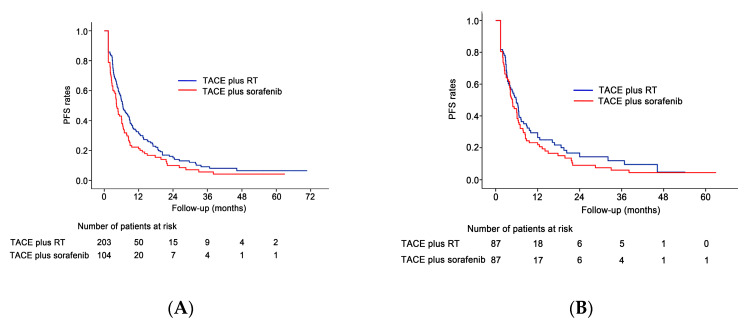
PFS outcomes before and after propensity score matching. (**A**) The PFS rate was significantly higher in the TACE plus RT group than in the TACE plus sorafenib group (*p* = 0.017) in the original study cohort. (**B**) The PFS rate did not differ significantly between the two groups (*p* = 0.258) in the propensity matched cohort.

During follow up, 161 patients (79.3%) in the TACE plus RT group and 92 (88.4%) in the TACE plus sorafenib group died or experienced HCC progression. The overall PFS rate was significantly higher in the TACE plus RT group than in the TACE plus sorafenib group (median survival, 6.5 vs. 4.3 months, respectively; *p* = 0.017; Figure 3A). In the TACE plus RT group, 58 patients (28.6%, 58/203) switched their treatment to other treatments (sorafenib monotherapy (*n* = 52), TACE plus sorafenib (*n* = 4), lenvatinib (*n* = 2)) owing to disease progression or complication of TACE, while 30 (28.8%, 30/104) in the TACE plus sorafenib group switched their treatment to other treatments (sorafenib monotherapy (*n* = 13), TACE monotherapy (*n* = 8), TACE plus RT (*n* = 5), nivolumab (*n* = 2), regorafenib (*n* = 2)) (*p* > 0.999) owing to disease progression or complication of TACE or sorafenib. In the propensity score-matched population, 70 patients (80.5%) in the TACE plus RT group and 77 (88.5%) in the TACE plus sorafenib group died or experienced HCC progression. After propensity score matching, no statistically significant difference in PFS was seen between the TACE plus RT and TACE plus sorafenib groups (median survival, 5.9 vs. 4.8 months, respectively; *p* = 0.258; Figure 3B).

### 3.4. Overall Survival Analyses

**Figure 4:**
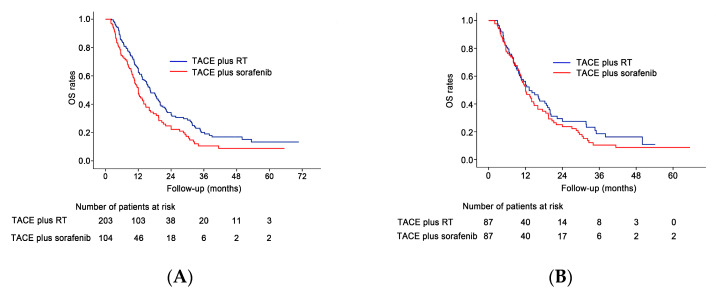
OS outcomes before and after propensity score matching. (**A**) The OS rate was significantly higher in the TACE plus RT group than in the TACE plus sorafenib group (*p* = 0.007) in the original study cohort. (**B**) The OS rate showed no statistically significant difference between the two groups (*p* = 0.299) in the propensity matched cohort.

By the end of the follow-up period, 130 patients (64%) in the TACE plus RT group and 84 (80.8%) in the TACE plus sorafenib group had died. The median survival times in the TACE plus RT and TACE plus sorafenib groups were 16.4 months (95% confidence interval (CI), 13.8–18.9 months) and 12 months (95% CI, 10.2–13.7 months), respectively. The cumulative OS rates at 1 and 3 years were 65.3% and 19% in the TACE plus RT group, and 50% and 10.5% in the TACE plus sorafenib group, respectively. The OS rates were significantly higher in the TACE plus RT group than in the TACE plus sorafenib group (*p* = 0.007; Figure 4A). In the propensity score-matched cohort, 60 (69%) of the TACE plus RT group patients and 71 (71.6%) of the TACE plus sorafenib group patients died. The cumulative OS rates in the propensity score-matched cohort at 1 and 3 years were 53.6% and 18.6% in the TACE plus RT group, and 50% and 10.4% in the TACE plus sorafenib group, respectively. After propensity score matching, no statistically significant difference in OS rate was seen between the TACE plus RT and TACE plus sorafenib groups (median survival, 13.2 vs.12 months, respectively; *p* = 0.299; Figure 4B).

### 3.5. Multivariable Analyses

Multivariable Cox regression analysis revealed that the adjusted hazard ratio (HR) for PFS and OS showed no significant association with the treatment type (Table 2). Covariates significantly associated with PFS and OS were tumor number, maximal tumor size, Child–Pugh class, and the presence or absence of extrahepatic metastasis.

### 3.6. Subgroup Analyses

**Figure 5:**
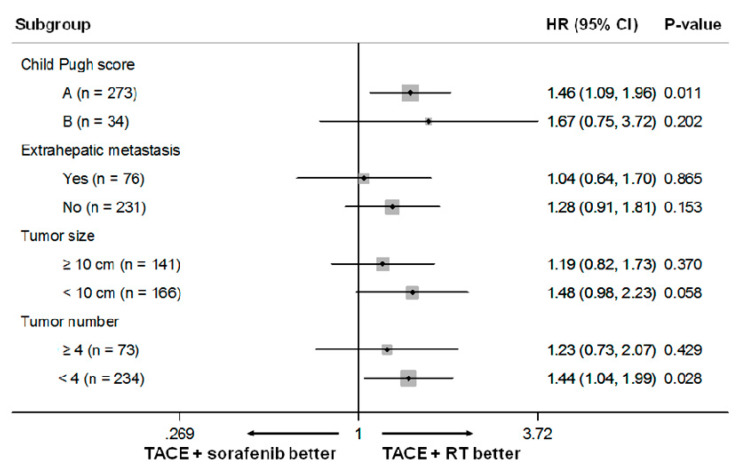
Forest plot of the treatment effect on OS in the subgroup analyses. The size of the squares is proportional to the size of the subgroups. Horizontal lines represent 95% confidence intervals. The position of each square represents the point estimate of the treatment effect.

Stratified subgroup analyses were performed according to the significant predictors for OS identified in the multivariable analysis (Figure 5). TACE plus sorafenib treatment was associated with a significantly poorer OS than TACE plus RT treatment in the subgroup of patients with Child–Pugh class A (HR, 1.46; 95% CI, 1.08–1.95; *p* = 0.011) and tumor number < 4 (HR, 1.44; 95% CI, 1.04–1.99; *p* = 0.028). The TACE plus sorafenib treatment showed poorer OS than the TACE plus RT in the subgroup of patients with maximal tumor size < 10 cm, with borderline statistical difference (HR, 1.48; 95% CI, 0.98–2.23; *p* = 0.058).

In the subgroup analysis for patients after excluding those with extrahepatic metastasis, the TACE plus sorafenib treatment showed poorer PFS (HR, 1.35; 95% CI, 0.98–1.86; *p* = 0.071) and OS (HR, 1.42; 95% CI, 1.00–2.03; *p* = 0.05) than the TACE plus RT with borderline statistical difference (Table 3).

### 3.7. Major Complications (Grade 3 or 4 Toxicities)

Major complications were observed in 19 (9.4%) patients in the TACE plus RT group and 14 patients (13.5%) in the TACE plus sorafenib group (*p* = 0.330). The major complications in the TACE plus RT group were grade 3 liver abscess (*n* = 5), grade 3 hepatic failure (*n* = 3), grade 3 anaphylaxis (*n* = 3), grade 3 hyperbilirubinemia (*n* = 2), grade 3 ileus (*n* = 1), grade 3 peritonitis (*n* = 1), grade 3 acute spinal cord infarction (*n* = 1), grade 4 ileus (*n* = 1), grade 4 acute kidney injury (*n* = 1), and grade 4 septic shock (*n* = 1). The major complications in the TACE plus sorafenib group were grade 3 hepatic failure (*n* = 3), grade 3 hyperbilirubinemia (*n* = 2), grade 3 nausea (*n* = 2), grade 3 diarrhea (*n* = 2), grade 3 liver abscess (*n* = 1), grade 3 thrombocytopenia (*n* = 1), grade 3 hand–foot skin reaction (*n* = 1), grade 3 general weakness (*n* = 1), and grade 3 acute respiratory distress syndrome (*n* = 1).

## 4. Discussion

This propensity score matching study showed no superiority between two combined strategies for treating patients with advanced HCC involving the PV. After propensity score matching, the median PFS and OS did not differ significantly between the TACE plus RT and TACE plus sorafenib groups. The multivariable analysis also found no significant association between PFS or OS and treatment type. However, in the subgroup analyses, TACE plus RT showed better outcomes in terms of OS in patients with limited tumor burden (≤3 tumors; maximal tumor size < 10 cm) and with preserved liver function (Child–Pugh class A). Although the main target of RT was PVTT, a considerable proportion of the tumor volume could be covered by RT if the patient had a minor tumor burden, which could be more effective than the combined treatment of TACE plus RT for patients with a major tumor burden. In addition, a sufficient dose of RT can be used if the patients have preserved liver function (Child–Pugh class A). By contrast, a recent meta-analysis found that the effectiveness and maintenance of sorafenib treatment was not significantly associated with the patient’s underlying liver function [25]. In the current study, both combined treatment strategies showed acceptable efficacy in prolonging survival in patients with HCC invading the PV, although TACE plus RT may be preferable in patients with limited tumor burden and preserved liver function. Further, for patients without extrahepatic metastasis, TACE plus RT tended to confer an OS benefit with borderline statistical difference in our subgroup analysis. Interestingly, we also found that TACE plus RT targeting only lesions within the liver was not associated with inferior survival compared to TACE plus sorafenib in the subset of patients with extrahepatic disease. This finding may support the prognostic significance of intrahepatic control even in HCC patients with additional metastatic tumors, which has been previously reported in different settings [26,27].

Currently, three randomized phase III trials of combined treatment (TACE plus sorafenib or TACE plus RT) have been conducted in patients with unresectable HCC. First, Kudo et al. tested the effectiveness of TACE plus sorafenib (*n* = 80) vs. TACE monotherapy (*n* = 76) in patients with unresectable HCC without vascular invasion and extrahepatic metastasis (mostly BCLC A and B patients, ≥80% in each group) [12]. In their study, the median PFS was significantly higher in the TACE plus sorafenib group than in the TACE monotherapy group (25.2 vs. 13.5 months, respectively; *p* = 0.006). OS was not evaluated in their study because an insufficient number of OS events were reached. Second, Park et al. conducted a randomized study to compare the efficacy of TACE plus sorafenib (*n* = 170) with sorafenib monotherapy (*n* = 169) in patients with unresectable HCC (mostly BCLC C patients, ≥ 70% in each group) [11]. Although the median PFS was significantly higher in the TACE plus sorafenib group (5.2 vs. 3.6 months vs. monotherapy; *p* = 0.003), the median OS showed no statistically significant difference between the two groups (12.8 vs. 10.8 months, respectively; *p* = 0.290). However, for patients with major (lobar or main) PVTT or any other vascular invasion, TACE plus sorafenib tended to confer an OS benefit with borderline statistical significance (HR, 0.52; 95% CI, 0.27–1.02; *p* < 0.1) [11]. In the propensity matched cohort of the current study, the median PFS and OS (4.8 and 12 months, respectively) of TACE plus sorafenib were very similar to those reported by Park et al. [11]. Third, a recent randomized controlled trial conducted by Yoon et al. found that TACE plus RT therapy (*n* = 45) offered a clear benefit in terms of PFS (7 vs. 2.6 months with sorafenib monotherapy (*n* = 45); *p* < 0.001) and OS (12.8 vs. 10 months with monotherapy; *p* = 0.04) in patients with HCC and macrovascular invasion [15]. The median PFS and OS associated with the TACE plus RT group in the current propensity matched cohort are also comparable to the results of Yoon et al. [15]. To date, there has been no randomized controlled trial of these two combined therapies for patients with HCC and PVTT. 

With introduction of immunotherapy for HCC, most recent studies on the combination of immune checkpoint inhibitor (such as pembrolizumab, atezolizumab, nivolumab, tremelimumab, durvalumab) and anti-angiogenic therapy (such as lenvatinib, bevacizumab) has shown synergic anti-tumor effects in patients with advanced HCC [28,29,30] and may improve PFS and OS as first-line treatment for advanced HCC. For instance, the phase 3 IMbrave 150 trial demonstrated statistically significant and clinically meaningful improvement in both OS and PFS for atezolizumab plus bevacizumab versus sorafenib in patients with unresectable HCC who have not received prior systemic therapy [28].

In addition, combination therapy of immune check inhibitor and locoregional treatment (such as radiofrequency ablation, TACE, radioembolization) is now actively investigated as a therapy of new paradigm for the treatment of HCC [31,32]. Duffy et al. [31] tested the synergic effect of tremelimumab in combination with tumor ablation for advanced HCC; ablation therapy induced a peripheral immune response which may enhance the effect of anti-cytotoxic T-lymphocyte-associated protein 4 treatment (tremelimumab). The results were promising; the median time to tumor progression and OS was 7.4 months and 12.3 months, respectively. To date, multiple trials on TACE plus pembrolizumab (NCT03397654, NCT03099564) and TACE plus nivolumab (NCT03143270, NCT03572582, NCT04268888) combination therapy are now recruiting patients. In addition, a trial (NCT03778957) on TACE in combination with durvalumab and bevacizumab is also now recruiting patients as the literature suggests a good synergistic effect of immune checkpoint inhibitor and anti-angiogenic therapy. Further studies can elucidate the best adjunct therapeutic options with locoregional therapies.

The limitations of the current study include its retrospective design and non-randomized nature, which make it vulnerable to a variety of potential biases. A prospective randomized controlled trial may provide a more definitive answer to the question of whether TACE plus RT and TACE plus sorafenib are equivalent or not in this specific group of patients. However, potential bias was minimized by basing the analysis on propensity score estimates in the current study [19]. A further limitation is that this is a single-center study, which may limit the generalizability of the results. Therefore, it is possible that our results may not be pertinent for patients with HCC in other countries, owing to differences in demographics and the underlying causes of liver disease [33].

## 5. Conclusions

In conclusion, in this retrospective study of patients with advanced HCC with PVTT, PFS and OS did not differ significantly between the TACE plus RT and TACE plus sorafenib groups. However, in the subgroup analysis, TACE plus RT was superior to TACE plus sorafenib, with borderline statistical significance in non-metastatic HCC patients.

## Figures and Tables

**Table 1:** Baseline patient characteristics.

	Study Population before PSM			Study Population after PSM		
Variable	TACE + Sorafenib	TACE + RT	*p* Value	TACE + Sorafenib	TACE + RT	*p* Value
Patients	104	203		87	87	
Age (years old, mean ± SD)	56.4 ± 10.8	55.6 ± 9.3	0.485	56.1 ± 10.6	55.8 ± 9.9	0.883
Male sex, *n* (%)	91 (87.5)	175 (86.2)	0.753	75 (86.2)	76 (87.4)	0.823
Etiology, *n* (%)			0.002			0.889
HBV	82 (78.8)	188 (92.6)		75 (86.2)	7 (88.5)	
HCV	9 (8.7)	6 (3)		4 (4.6)	3 (3.4)	
Others	13 (12.5)	9 (4.4)		8 (9.2)	7 (8)	
ECOG PS			0.747			0.564
0	63 (59.6)	112 (55.2)		52 (59.8)	55 (63.2)	
1	41 (38.5)	86 (42.4)		34 (39.1)	32 (36.8)	
2	2 (1.9)	5 (2.5)		1 (1.1)	0 (0)	
Child–Pugh score, *n* (%)			0.569			0.635
A	91 (87.5)	182 (89.7)		76 (87.4)	78 (89.7)	
B	13 (12.5)	21 (10.3)		11 (12.6)	9 (10.3)	
Presence of extrahepatic metastasis	39 (37.5)	37 (18.2)	< 0.001	29 (33.3)	25 (28.7)	0.512
Maximal tumor size (cm, mean ± SD)	10.6 ± 4.2	9.2 ± 4.0	0.003	10.2 ± 4.1	10.1 ± 4.0	0.762
Tumor number ≥ 4	31 (29.8)	42 (20.7)	0.076	21 (24.1)	20 (23)	0.858
Bilirubin (mg/dL, mean ± SD)	0.91 ± 0.49	0.90 ± 0.50	0.843	0.90 ± 0.50	0.88 ± 0.58	0.846
Albumin (≤ 3.5 mg/dL), *n* (%)	52 (50)	76 (37.4)	0.038	38 (43.7)	40 (46)	0.76
AFP ≥400 ng/mL, *n* (%)	60 (57.7)	113 (55.7)	0.735	49 (56.3)	49 (56.3)	> 0.999
Tumor type, *n* (%)			0.678			0.436
Nodular	42 (40.4)	87 (42.9)		36 (41.4)	31 (35.6)	
Infiltrative	62 (59.6)	116 (57.1)		51 (58.6)	56 (64.4)	
Tumor involvement, *n* (%)			0.629			0.609
Unilobar	71 (68.3)	133 (65.5)		62 (71.3)	65 (74.7)	
Bilobar	33 (31.7)	70 (34.5)		25 (28.7)	22 (25.3)	
Extent of PVTT			0.102			0.803
Main portal vein	12 (11.6)	20 (9.9)		9 (10.4)	9 (10.4)	
First-order portal vein branch	59 (56.7)	93 (45.8)		47 (54)	51 (58.6)	
Second- or lower-order portal vein branches	33 (31.7)	90 (44.3)		31 (35.6)	27 (31)	
Combined hepatic vein invasion	19 (18.3)	24 (11.8)	0.123	14 (16.1)	16 (18.4)	0.688

PSM, propensity score matching; TACE, transarterial chemoembolization; RT, radiotherapy; SD, standard deviation; *n*, number; HBV, hepatitis B virus; HCV, hepatitis C virus; ECOG PS, Eastern Cooperative Oncology Group performance status; PVTT, portal vein tumor thrombosis; AFP, alpha-fetoprotein.

**Table 2:** Results of Cox proportional hazard models.

Analysis		Hazard Ratio	95% Confidence Interval		*p* Value
PFS					
Unadjusted	TACE plus sorafenib	1.354	1.048–1.751		0.021
	TACE plus RT	1			
Adjusted †	TACE plus sorafenib	1.015	0.766–1.245		0.917
	TACE plus RT	1			
Propensity matched *	TACE plus sorafenib	1.2	0.88–1.635		0.248
	TACE plus RT	1			
OS					
Unadjusted	TACE plus sorafenib	1.451	1.102–1.909		0.008
	TACE plus RT	1			
Adjusted ‡	TACE plus sorafenib	1.164	0.874–1.551		0.299
	TACE plus RT	1			
Propensity matched *	TACE plus sorafenib	1.199	0.865–1.661		0.276
	TACE plus RT	1			

PFS, progression-free survival; TACE, transarterial chemoembolization; RT, radiotherapy; OS, overall survival. † Adjusted for tumor number, maximal tumor size, tumor extent, serum bilirubin level, Child–Pugh class, and presence of extrahepatic metastasis, which were significant in the univariable analysis. * Cox proportional hazard models, with robust standard errors that accounted for the clustering of matched pairs. ‡ Adjusted for tumor number, maximal tumor size, tumor extent, serum bilirubin level, serum albumin level, Child–Pugh class, ECOG performance status, and presence of extrahepatic metastasis, which were significant in the univariable analysis.

**Table 3:** Results of Cox proportional hazard models in subgroup without extrahepatic metastasis.

Analysis		Hazard Ratio	95% Confidence Interval		*p* Value
PFS					
Unadjusted	TACE plus sorafenib	1.271	0.925–1.747		0.139
	TACE plus RT	1			
Adjusted †	TACE plus sorafenib	1.345	0.975–1.856		0.071
	TACE plus RT	1			
OS					
Unadjusted	TACE plus sorafenib	1.285	0.909–1.815		0.155
	TACE plus RT	1			
Adjusted ‡	TACE plus sorafenib	1.424	1.001–2.026		0.05
	TACE plus RT	1			

PFS, progression-free survival; TACE, transarterial chemoembolization; RT, radiotherapy; OS, overall survival. †Adjusted for tumor number, maximal tumor size, tumor extent, serum bilirubin level, and Child–Pugh class, which were significant in the univariable analysis. ‡ Adjusted for tumor number, maximal tumor size, and Child–Pugh class, which were significant in the univariable analysis.

## References

[1] Llovet J.M., Ricci S., Mazzaferro V., Hilgard P., Gane E., Blanc J.F., de Oliveira A.C., Santoro A., Raoul J.L., Forner A. (2008). Sorafenib in advanced hepatocellular carcionoma. N. Engl. J. Med..

[2] Cheng A.L., Kang Y.K., Chen Z., Tsao C.J., Qin S., Kim J.S., Luo R., Feng J., Ye S., Yang T.S. (2009). Efficacy and safety of sorafenib in patients in the Asia-Pacific region with advanced hepatocellular carcionoma: A phase III randomised, double-blind, placebo-controlled trial. Lancet Oncol..

[3] Bruix J., Cheng A.L., Meinhardt G., Nakajima K., De Sanctis Y., Llovet J. (2017). Prognostic factors and predictors of sorafenib benefit in patients with hepatocellular carcinoma: Analysis of two phase III studies. J. Hepatol..

[4] Llovet J.M., Bustamante J., Castells A., Vilana R., Ayuso Mdel C., Sala M., Bru C., Rodes J., Bruix J. (1999). Natural history of untreated nonsurgical hepatocellular carcinoma: Rationale for the design and evaluation of therapeutic trials. Hepatology.

[5] Schöniger-Hekele M., Müller C., Kutilek M., Oesterreicher C., Ferenci P., Gangl A. (2001). Hepatocellular carcinoma in Central Europe: Prognostic features and survival. Gut.

[6] Cabibbo G., Enea M., Attanasio M., Bruix J., Craxì A., Cammà C. (2010). A meta-analysis of survival rates of untreated patients in randomized clinical trials of hepatocellular carcinoma. Hepatology.

[7] Chung G.E., Lee J.H., Kim H.Y., Hwang S.Y., Kim J.S., Chung J.W., Yoon J.H., Lee H.S., Kim Y.J. (2011). Transarterial chemoembolization can be safely performed in patients with hepatocellular carcinoma invading the main portal vein and may improve the overall survival. Radiology.

[8] Kim K.M., Kim J.H., Park I.S., Ko G.Y., Yoon H.K., Sung K.B., Lim Y.S., Lee H.C., Chung Y.H., Lee Y.S. (2009). Reappraisal of repeated transarterial chemoembolization in the treatment of hepatocellular carcinoma with portal vein invasion. J. Gastroenterol. Hepatol..

[9] Kim J.H., Shim J.H., Yoon H.K., Ko H.K., Kim J.W., Gwon D.I. (2018). Chemoembolization related to good survival for selected patients with hepatocellular carcinoma invading segmental portal vein. Liver Int..

[10] Kim G.A., Shim J.H., Yoon S.M., Jung J., Kim J.H., Ryu M.H., Ryoo B.Y., Kang Y.K., Lee D., Kim K.M. (2015). Comparison of chemoembolization with and without radiation therapy and sorafenib for advanced hepatocellular carcinoma with portal vein tumor thrombosis: A propensity score analysis. J. Vasc. Interv. Radiol..

[11] Park J.W., Kim Y.J., Kim D.Y., Bae S.H., Paik S.W., Lee Y.J., Kim H.Y., Lee H.C., Han S.Y., Cheong J.Y. (2019). Sorafenib with or without concurrent transarterial chemoembolization in patients with advanced hepatocellular carcinoma: The phase III STAH trial. J. Hepatol..

[12] Kudo M., Ueshima K., Ikeda M., Torimura T., Tanabe N., Aikata H., Izumi N., Yamasaki T., Nojiri S., Hino K. (2019). Randomised, multicentre prospective trial of transarterial chemoembolisation (TACE) plus sorafenib as compared with TACE alone in patients with hepatocellular carcinoma: TACTICS trial. Gut.

[13] Yoon S.M., Lim Y.S., Won H.J., Kim J.H., Kim K.M., Lee H.C., Chung Y.H., Lee Y.S., Lee S.G., Park J.H. (2012). Radiotherapy plus transarterial chemoembolization for hepatocellular carcinoma invading the portal vein: Long-term patient outcomes. Int. J. Radiat. Oncol. Biol. Phys..

[14] Koo J.E., Kim J.H., Lim Y.S., Park S.J., Won H.J., Sung K.B., Suh D.J. (2010). Combination of transarterial chemoembolization and three-dimensional conformal radiotherapy for hepatocellular carcinoma with inferior vena cava tumor thrombus. Int. J. Radiat. Oncol. Biol. Phys..

[15] Yoon S.M., Ryoo B.Y., Lee S.J., Kim J.H., Shin J.H., An J.H., Lee H.C., Lim Y.S. (2018). Efficacy and safety of transarterial chemoembolization plus external beam radiotherapy vs sorafenib in hepatocellular carcinoma with macroscopic vascular invasion: A randomized clinical trial. JAMA Oncol..

[16] European Association For The Study Of The Liver (2018). EASL Clinical Practice Guidelines: Management of hepatocellular carcinoma. J. Hepatol..

[17] Marrero J.A., Kulik L.M., Sirlin C.B., Zhu A.X., Finn R.S., Abecassis M.M., Roberts L.R., Heimbach J.K. (2018). Diagnosis, Staging, and Management of Hepatocellular carcinoma: 2018 Practice Guidance by the American Association for the Study of Liver Diseases. Hepatology.

[18] Kim Y.J., Jung J., Joo J.H., Kim S.Y., Kim J.H., Lim Y.S., Lee H.C., Kim J.H., Yoon S.M. (2019). Combined transarterial chemoembolization and radiotherapy as a first-line treatment for hepatocellular carcinoma with macroscopic vascular invasion: Necessity to subclassify Barcelona Clinic Liver Cancer stage C. Radiother. Oncol..

[19] Choi G.H., Shim J.H., Kim M.J., Ryu M.H., Ryoo B.Y., Kang Y.K., Shin Y.M., Kim K.M., Lim Y.S., Lee H.C. (2013). Sorafenib alone versus sorafenib combined with transarterial chemoembolization for advanced-stage hepatocellular carcinoma: Results of propensity score analyses. Radiology.

[20] Zhu K., Chen J., Lai L., Meng X., Zhou B., Huang W., Cai M., Shan H. (2014). Hepatocellular carcinoma with portal vein tumor thrombus: Treatment with transarterial chemoembolization combined with sorafenib—A retrospective controlled study. Radiology.

[21] Lencioni R., Llovet J.M. (2010). Modified RECIST (mRECIST) assessment for hepatocellular carcinoma. Semin. Liver Dis..

[22] Lencioni R., Montal R., Torres F., Park J.W., Decaens T., Raoul J.L., Kudo M., Chang C., Rios J., Boige V. (2017). Objective response by mRECIST as a predictor and potential surrogate end-point of overall survival in advanced HCC. J. Hepatol..

[23] Saad E.D., Katz A. (2009). Progression-free survival and time to progression as primary end points in advanced breast cancer: Often used, sometimes loosely defined. Ann. Oncol..

[24] Ju M.J., Tu G.W., Han Y., He H.Y., He Y.Z., Mao H.L., Wu Z.G., Yin Y.Q., Luo J.F., Zhu D.M. (2013). Effect of admission time on mortality in an intensive care unit in Mainland China: A propensity score matching analysis. Crit. Care.

[25] McNamara M.G., Slagter A.E., Nuttall C., Frizziero M., Pihlak R., Lamarca A., Tariq N., Valle J.W., Hubner R.A., Knox J.J. (2018). Sorafenib as first-line therapy in patients with advanced Child-Pugh B hepatocellular carcinoma—A meta-analysis. Eur. J. Cancer.

[26] Bettinger D., Spode R., Glaser N., Buettner N., Boettler T., Neumann-Haefelin C., Brunner T.B., Gkika E., Maruschke L., Thimme R. (2017). Survival benefit of transarterial chemoembolization in patients with metastatic hepatocellular carcinoma: A single center experience. BMC Gastroenterol..

[27] Kim J., Sinn D.H., Choi M.S., Kang W., Gwak G.Y., Paik Y.H., Lee J.H., Koh K.C., Paik S.W. (2019). Hepatocellular carcinoma with extrahepatic metastasis: Are there still candidates for transarterial chemoembolization as an initial treatment?. PLoS ONE.

[28] Cheng A.L., Qin S., Ikeda M., Galle P., Ducreux M., Zhu A., Kim T.Y., Kudo M., Breder V., Merie P. (2019). IMbrave 150: Efficacy and safety results from a ph III study evaluating atezolizumab (atezo) + bevacizumab (bev) vs sorafenib (Sor) as first treatment (tx) for patients (pts) with unresectable hepatocellular carcinoma (HCC). Ann. Oncol..

[29] Stein S., Pishvaian M.J., Lee M.S., Lee K.H., Hernandez S., Kwan A., Liu B., Grossman W., Iizuka K., Ryoo B.Y. (2018). Safety and clinical activity of 1L atezolizumab + bevacizumab in a phase Ib study in hepatocellular carcinoma (HCC). J. Clin. Oncol..

[30] Ikeda M., Sung M.W., Kudo M., Kobayashi M., Baron A.D., Finn R.S., Kaneko S., Zhu A.X., Kubota T., Kraljevic S. (2018). A Phase 1B trial of lenvatinib (LEN) plus pembrolizumab (PEM) in patients (PTS) with unresectable hepatocellular carcinoma (uHCC). J. Clin. Oncol..

[31] Duffy A.G., Ulahannan S.V., Makorova-Rusher O., Rahma O., Wedemeyer H., Pratt D., Davis J.L., Hughes M.S., Heller T., ElGindi M. (2017). Tremelimumab in combination with ablation in patients with advanced hepatocellular carcinoma. J. Hepatol..

[32] Zhan C., Ruohoniemi D., Shanbhogue K.P., Wei J., Welling T.H., Gu P., Park J.S., Dagher N.N., Taslakian B., Hickey R.M. (2020). Safety of combined yttrium-90 radioembolization and immune checkpoint inhibitor immunotherapy for hepatocellular carcinoma. J. Vasc. Interv. Radiol..

[33] Chu H.H., Kim J.H., Kim P.N., Kim S.Y., Lim Y.S., Park S.H., Ko H.K., Lee S.G. (2019). Surgical resection versus radiofrequency ablation very early-stage HCC (</=2 cm Single HCC): A propensity score analysis. Liver Int..

